# Preparation of cleared whole-mount urethra and urinary bladder from transcardially perfused mice for immunolabeling and analysis by CLSM

**DOI:** 10.1016/j.xpro.2026.104542

**Published:** 2026-05-07

**Authors:** Uwe Pfeil, Felix Dettler, Alexander Perniss, Praju Vikas Anekal, Lora Bankova, Paula Montero LIopis, Klaus Deckmann

**Affiliations:** 1 Institute for Anatomy and Cell Biology, Justus-Liebig-University, Giessen, Germany; 2 Division of Allergy and Clinical Immunology, Brigham and Women’s Hospital and Department of Medicine, Harvard Medical School, Boston, MA, USA; 3 MicRoN Core, Harvard Medical School, Boston, MA, USA

**Keywords:** Cell Biology, Microscopy

## Abstract

The distribution of cells within organs, their involvement in tissue structure, and their organization in three-dimensional networks are important aspects in the analysis and understanding of biological processes. Here, we present a protocol for preparing whole-mount urethra and urinary bladder from transcardially perfused mice for immunolabeling and analysis by confocal laser scanning microscopy. We describe steps for perfusion, preparation, tissue clearing, and analysis using confocal laser scanning microscopy. We then detail procedures for three-dimensional processing of the murine urethra and urinary bladder.

For complete details on the use and execution of this protocol, please refer to Schmidt et al.^1^

## Highlights

• Instructions for the transcardial perfusion of a mouse
• Steps for preparation of male and female urethrae and bladders
• Guidance on tissue clearing, immunohistochemistry, and confocal laser scanning microscopy
• Procedures for 3D rendering

## Abstract (editor highlights)

Publisher’s note: Undertaking any experimental protocol requires adherence to local institutional guidelines for laboratory safety and ethics.

## Graphical abstract

**Figure:**
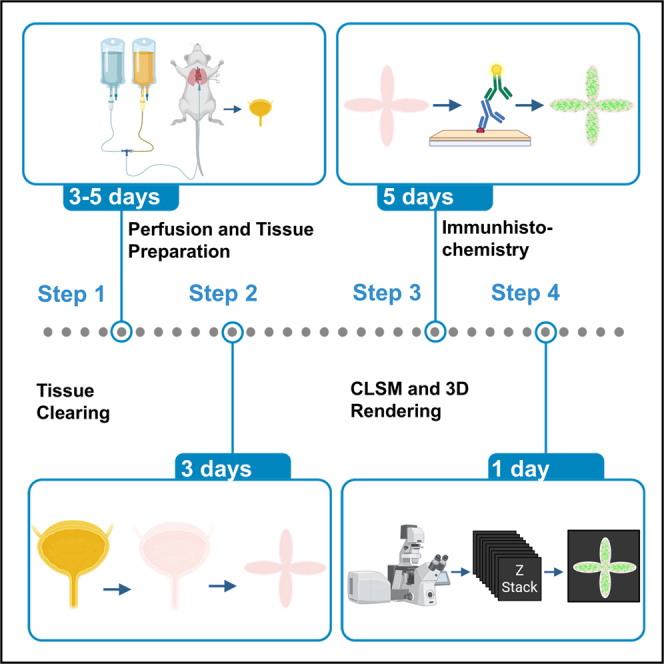

## Before you begin

The protocol below describes a specific method for producing images and videos of optical cleared tissue samples, using organs of the murine urogenital tract. However, the method can also be used for other organs. It should be noted that the method has to be adapted to other organs of interest and that differences may occur depending on the composition and thickness of the organ desired, e.g., with regard to incubation times. Tissue clearing is an excellent method to study cells and a simple and elegant way to obtain a 3D model of the cellular environment within an organ.

The first essential step for the successful implementation of this method is fixation of the organs by perfusion, which preserves the organs and remove interfering factors such as erythrocytes. The organ can then be removed, cleared and examined using confocal laser scanning microscopy (CLSM). The combination of these three techniques provides the possibility for 3D analysis of cellular structures.

### Innovation

We provide a detailed protocol, from perfusion and tissue clearing up to analysis using confocal laser scanning microscopy and three-dimensional processing. Our perfusion protocol is based on the protocol developed by Forssmann et al. 1977 adapted and modified for transcardial perfusion.^2^ For fixation we use Zamboni solution, a phosphate-buffered picric acid–formaldehyde (PAF) fixative designed for application in light and electron microscopy. Zamboni fixative ensures rapid penetration, effective general fixation, and optimal preservation and stabilization of cellular proteins. The clearing protocol is based on the work of Treweek et al. 2015 adapted and modified for urethra and urinary bladder.^3^ Samples were subsequently suitable for CLSM analysis and 3D rendering. With this, we adapted, modified, and merged these steps into a workflow to provide a coordinated protocol.

### Institutional permissions (if applicable)

All animal experiments describe here were carried out in accordance with the recommendations of the European Communities Council Directive of 24^th^ November 1986 (86/609/EEC) and performed in accordance with the German animal welfare law and had been declared to the Animal Welfare Officer of the University (Registration No. 793_M, 840_M). All experiments conform to the relevant regulatory standards. The method used for “humane killing” the animals were consistent with the recommendations of the AVMA Guidelines for the Euthanasia of Animals.

### Protocol

**Timing: 5 h**

#### Preparation of pre-rinse solution

Pre-rinse solution contains polyvinylpyrrolidone K30 (PVP), sodium chloride (NaCl), procaine and heparin. It is used to wash blood out of vessels prior fixation.

1. Preparing Pre-rinse solution.
  a. Dissolve 25 g PVP (2.5% final concentration), 9 g NaCl (0.9% final concentration) and 5 g procaine hydrochloride (0.5% final concentration) in ddH_2_O to 1000 mL in a 1.5 l Erlenmeyer flask.
    ***Note:*** Approximately 200 mL pre-rinse solution is required for one animal.
  b. Adjust the pH value to 7.3–7.4 using sodium hydroxide.
  c. Mix solution for approximal 4 h on a magnetic stirrer.
    ***Note:*** These chemicals are difficult to dissolve in water, so this step takes some time.
  d. Pre-rinse solution could be stored for up to 4 days at 2°C–8°C or kept frozen at −20°C.
    ***Note:*** Storage should be in suitable plastic containers. Thawing the solution takes some time and should be started 24 h before the start of the experiment.
  e. Pre-rinse solution should be pre-warmed to body temperature to enhance flow and chemical reactivity.
  f. Right before use add 2 mL heparin-sodium solution (25.000 I.E./5 mL; 10000 I.E./l final concentration).
    **CRITICAL:** Heparin-sodium should not be added in advance, because of possible adherence to the storage container wall.

### Preparation of Zamboni fixation solution

**Timing: 3 h**

For fixation we use Zamboni solution∗ (2% paraformaldehyde/15% saturated picric acid in 0.1 M phosphate buffer, pH 7.4). Zamboni solution is a phosphate-buffered picric acid–formaldehyde (PFA) fixative designed for application in light and electron microscopy. It ensures rapid penetration, effective general fixation, and optimal preservation and stabilization of cellular proteins. At this point, we would like to mention that we have found that some antibodies only work on tissue fixed with Zamboni.

2. Preparing saturated picric acid.
  a. Dissolve 50 g of picric acid in 1000 mL ddH_2_O.
  b. Leave the solution to stand for 8 h.
    ***Note:*** This will result in formation of a crystalline sediment. The saturated picric acid remains as supernatant.
  c. Filtrate the solution twice though a pleated filter and keep the filtrate.
    **CRITICAL:** Picric acid is an organic compound of yellow color. It is a potent explosive when dry but can be handled more safely when kept wet with at least 30% water, which desensitizes it to shock and heat. Therefore, picric acid moistened with water, ≥98% should be purchased.
    ∗Harmful or toxic and explosive in a crystalline state; please refer to the safety data sheet (Supplementary Information 1).
3. Preparing phosphate buffer 0.1 M and 0.2 M.
  a. Preparing solution A: Dissolve 31.2 g sodium dihydrogen phosphate dihydrate to 1000 mL ddH_2_O water.
  b. Preparing solution B: Dissolve 35.6 g disodium hydrogen phosphate dihydrate to 1000 mL ddH_2_O water.
  c. Preparing 0.2 M phosphate buffer: Take 230 mL solution A, add 770 mL solution.
    ***Note:*** This yields 1 liters of 0.2 phosphate buffer.
  d. Preparing 0.1 M phosphate buffer: Take 230 mL solution A, add 770 mL solution B, add 1000 mL ddH_2_O water.
    ***Note:*** This yields 2 liters of 0.1 phosphate buffer.
  e. Adjust the pH value to 7.3–7.4 using sodium hydrochloric acid.
    ***Note:*** 0.2 phosphate buffer is necessary for preparation of Zamboni solution. 0.1 phosphate buffer is necessary for washing steps during tissue processing.
4. Preparing Zamboni solution.
  a. Take 50 mL of formaldehyde solution (37% in water; formalin).
  b. Add 500 mL 0.2 M phosphate buffer.
  c. Add 150 mL saturated picric acid.
  d. Mix solution on a magnetic stirrer.
  e. Add ddH_2_O water up to one liter.
  f. Zamboni solution should be pre-warmed to body temperature to enhance flow and chemical reactivity.

## Key resources table

**Table:** 

REAGENT OR RESOURCE	SOURCE	IDENTIFIER
**Antibodies**		

Sheep anti-Tyrosine Hydroxylase; 1:400	Millipore	AB1542 RRID:AB_90755
Chicken-anti-GFP (green fluorescent protein); 1:2000	Novus	Novus Cat# NB100-1614 RRID:AB_10001164
Rat-anti-substance P; 1:400	Santa Cruz	Santa Cruz Biotechnology Cat# sc-21715, RRID:AB_628299
Rabbit anti-CGRP; 1:20000	Peninsula	Peninsula Laboratories Cat# T-4032 RRID:AB_518147
Donkey IgG anti-Sheep IgG (H+L)-Cy3; 1:2000	Dianova	Jackson ImmunoResearch Labs Cat# 713-165-003, RRID:AB_2340727
Donkey IgG anti-chicken IgY (H+L), FITC-conjugate; 1:800	Dianova	Jackson ImmunoResearch Labs Cat# 703-095-155, RRID:AB_2340356
Donkey-anti-rat IgG Cy3; 1:1000	Dianova	(Jackson ImmunoResearch Labs Cat# 712-165-150, RRID:AB_2340666)
Donkey IgG anti-rabbit IgG (H+L), Cy5-conjugate; 1:400	Dianova	Jackson ImmunoResearch Labs Cat# 711-175-152, RRID:AB_2340607

**Chemicals, peptides, and recombinant proteins**		

Polyvinylpyrrolidone K30 (PVP)	Roth	Cat# 4607.1, CAS Nr. 9003-39-8
Sodium chloride (NaCl)	Roth	Cat# 5741.1 CAS Nr. 7647-14-5
Procaine hydrochloride	Merck	Cat# P9879-50G CAS Nr. 51-05-8
Milli-Q water	–	–
Heparin-sodium 25.000 I.E/5 mL	Panpharma	PZN16200037/EAN 4150162000373
Picric acid	Merck	Cat# 197378 CAS Nr. 88-89-1
Sodium dihydrogen phosphate dihydrate	Roth	Cat# T879.1 CAS No. 13472-35-0
Disodium hydrogen phosphate dihydrate	Roth	Cat# 4984.1 CAS No. 10028-24-7
Formaldehyde solution	Sigma-Aldrich	Cat# 252549 CAS No. 50-00-0
Isoflurane	Sigma-Aldrich	Cat# 792632 CAS No. 26675-46-7
PBS phosphate-buffered saline (1.06 mM KH_2_PO_4_, 155.2 mM NaCl, 2.9 mM Na_2_HPO_4_-7H_2_O)	Thermo Fischer Scientific, Waltham, MA, USA	–
10% horse serum	–	–
0.5% Tween 20	–	–
0.1% bovine serum	–	–
Histodenz (nonionic density gradient medium)	Merck	CAS 66108-95-0 MDL MFCD00077732
Acrylamide	SIGMA	CAS 79-06-1 MDL MFCD00008032
2,2′-azobis [2-(2-imidazolin-2-yl)propane] dihydrochloride	WAKO Chenicals	CAS 27776-21-2
SDS	Roth	CAS 151-21-3
Sodium azide	Merck	CAS 26628-22-8 MDL MFCD00003536

**Experimental models: Organisms/strains**		

Mouse: C57BL/6JRj	Janvier Labs	RRID:IMSR_RJ:C57BL-6JRJ
Mouse: B6.Cg-Tg(RP23-268L19-EGFP)2Mik/J (ChAT-eGFP)	The Jackson Laboratory	Stock No: 007902 RRID:IMSR_JAX:007902

**Software and algorithms**		

Biorender	Biorender	Biorender (RRID:SCR_018361)
ZEN 2010B SP1	Zeiss	ZEISS ZEN Microscopy Software (RRID:SCR_013672)
Leica Application Suite X	Leica	RRID:SCR_013673
Arivis Vision4D software (Version 4.1.2,).	Arivis	https://kb.arivis.com/ RRID:SCR_018000

**Other**		

Folded filter MN 616 ¼, Diameter: 18.5 cm	MACHEREY-NAGEL	MANA_Z0036 EAN: 4064343162906
Pyrex® Erlenmeyer flask, narrow neck, with printed trace code	Merck	SLW1130/05 M
BRAND® glass powder funnel short wide stem	Merck	BR146511
Duran® GLS 80 wide mouth graduated laboratory bottles with cap and pouring ring	Merck	Z674109
Magnetic Stirring bar	–	–
Magnetic Stirrer	–	–
Dissecting scissors, coarse	FST	14510–15
Dissecting scissors, medium	FST	14184–09
Dissecting scissors, fine 14 cm straight	FST	15020–15
Forceps, fine	FST	11252–50
Forceps, coarse	FST	11992–12
Clamp (artery clamp)	FST	13018–14
TAIJI-IE Small Animal Anesthesia Machine	RWD	TAIJI-IE
Infusion bottle (1000 mL, clear, Stopper Infusion 32 mm) or Infusion bottle, for example Braun’s isotonic sodium chloride solution 0.9%	Packsys Braun	10000263 PZN:08646871/EAN4030539036313
Glass-tubing cutter	Merck	Z150770-1EA
Pravaz needle with LL mount, blunt version, size 1 diameter 0.9 mm at least 38 mm length or Laboratory pipetting needle with 90° blunt ends, gauge 20, L 4 in., 304 stainless steel hub	Acufirm Merck	1400 LL CAD7950-12EA
Scienceware® trays; L × W × H 30 cm × 41 cm × 7.6 cm	Merck	Z503797-1EA
Stainless steel perforated sheet, square hole 10 mm - thickness 2.00 mm; L × W 31 cm × 42 cm	Elbemetal	www.elbemetall.de
Intrafix® SafeSet with LL mount 180 cm with 3 × 4.1 mm tubing	Braun	PZN 01900697/EAN 4022495869517
Syring 1 mL	Braun	PZN 00896456 EAN 4022495252074
Injection needle (pierce the filter) 21G×4 4/5; 0,8 × 120 mm	Braun	PZN 04291448/EAN 4022495052414
Injection needle (injection of Heparin) 26G×1; 0,45 × 25 mm	Braun	PZN 2050858 EAN 4022495001016
Metal multi-hook for glass infusion bottles	Braun	PZN 04800507/EAN 4022495260161
Infusion accessories Heidelberg extensions 75 cm	Braun	PZN 02133113/EAN 4022495004758
Braunostat® U Universal infusion stand	Braun	8727902
Discofix®-3 three-way tap with connecting pipe, 50 cm, blue	Braun	PZN 07128507/EAN 4022495005007
Zeiss LSM 710 Confocal Inverted Microscope	Zeiss	RRID:SCR_018063
Leica STELLARIS 5 Microscope	Leica	RRID:SCR_024663

## Materials and equipment

**Table:** Pre-rinse solution

Reagent	Final concentration	Amount
Polyvinylpyrrolidone K30^a^	2.5%	25 g
Sodium chloride	0.9%	9 g
Procaine hydrochloride^a^	0.5%	5 g
Heparin natrium 25.000 I.E./5 mL^a^	10000 I.E./l	2 mL
ddH_2_O	N/A	1000 mL
**Total**	**N/A**	**1000 mL**

Stored for up to 4 days at 2°C–8°C or freeze at −20°C.

a Harmful or toxic; handle with care.

**CRITICAL:** Add Heparin-sodium immediately before use.

**Table:** Zamboni solution^a^

Reagent	Final concentration	Amount
Formaldehyde solution^a^ (37%)	2%	50 mL
0.2 M phosphate buffer	0.1 M	500 mL
Saturated picric acid^a^	15%	150 mL
ddH_2_O	N/A	Add to 1000 mL
**Total**	**N/A**	**1000 mL**

Stored for at least 1 year at 2°C–8°C.

a Harmful or toxic and explosive in a crystalline state; handle with care, please refer to the safety data sheet (Supplementary Information 1). If there are concerns about handling picric acid, we would like to point out that Zamboni solution is also commercially available.

**Table:** Hydrogel solution

Reagent	Final concentration	Amount
Acrylamide^a^	4%	4 g
2,2′-azobis [2-(2-imidazolin-2-yl)propane] dihydrochloride	0.25%	250 mg
0.1 M phosphate buffer	N/A	Add to 100 mL
**Total**	**N/A**	**100 mL**

a Neurotoxic; handle with care.

***Note:*** It is important to precool phosphate buffer to 4°C before addition of acrylamide and 2,2′-azobis [2-(2-imidazolin-2-yl)propane] dihydrochloride.

**CRITICAL:** Prepare immediately before use.

**Table:** SDS solution

Reagent	Final concentration	Amount
SDS	8%	8 g
0.1 M phosphate buffer	N/A	Add to 100 mL
**Total**	**N/A**	**100 mL**

Stored for at least 1 year at 2°C–8°C.

**Table:** Refractive index matching solution (RIMS)

Reagent	Final concentration	Amount
Histodenz	80%	40 g
sodium azide	0.01%	5 mg
0.02 M phosphate buffer pH 7.5	N/A	Add to 50 mL
**Total**	**N/A**	**50 mL**

**CRITICAL:** Prepare immediately before use.

### Equipment

Various items are required for this protocol. Some of them are not commercially available and have to be prepared in advance.

### Perfusion bottles

**Figure 1:**
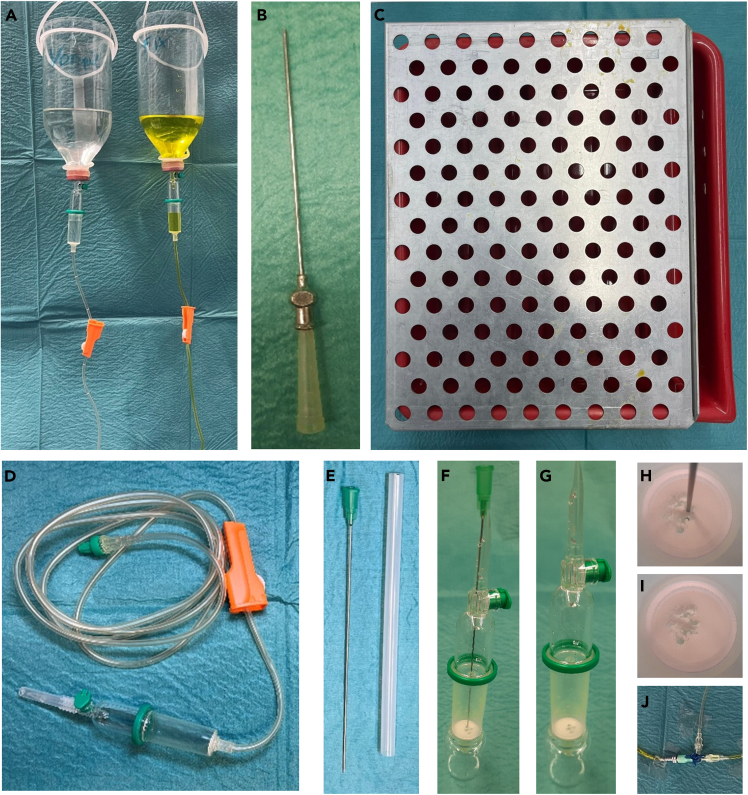
Materials and equipment (A) Perfusion bottles filled with prerinse solution (left) and yellow Zamboni fixation solution (right) connected to an Infrafix SafeSet. (B) Blunt perfusion needle (V2A steel pravaz needle with LL mount size 1 diameter 0.9 mm). (C) Perfusion table (perforated stainless-steel sheet with mounted on square plastic tray). (D) Infrafix SafeSet kits. (E) Injection needle 21G×4 4/5; 0.8 × 120 mm to perforate the filter in the Infrafix SafeSet kits. (F–I) Perforation of the filter in the Infrafix SafeSet kits using an injection needle. (J) 3-way tap.

Two perfusion bottles are required for perfusion: One for the pre-flush solution and one for the fixation solution (Figure 1A). Once prepared, they can be reused. We recommend glass bottles with rubber stoppers. It is necessary to remove the bottom of the bottles with a glass-tubing cutter. Alternatively, plastic infusion bottles without bottom, for example Braun’s isotonic sodium chloride solution 0.9%, can be used.

### Perfusion needle

For perfusion a blunt perfusion needle is needed. We recommend using a blunt V2A steel pravaz needle with LL mount size 1 diameter 0.9 mm at least 38 mm length. 0.9 mm corresponds to 20G (Figure 1B). Alternatively, a laboratory pipetting needle with 90° blunt end Gauge 20, L4 in. 304 stainless steel hub could be used.

### Perfusion table

We recommend the use of a perfusion table (Figure 1C). Therefore, a stainless-steel perforated sheet with square hole 10 mm and a thickness of 2.00 mm can be mounted on square plastic tub or tray. For practical purposes, the Scienceware® trays have a built-in drain, which makes disposing of chemicals much more convenient.

### Perfusion tubing

We recommend using two Infrafix SafeSet kits (Figure 1D). These Infrafix SafeSet kits contain a filter to retain coarse particles. Unfortunately this filter impedes perfusion and must therefore be pierced in advance with a long needle, e.g., Injection needle 21Gx4 4/5; 0.8 × 120 mm (Figure 1E). Otherwise, the fluid will not flow at the required pressure and speed, what may cause perfusion failure.

**CRITICAL:** Filter must be pierced in advance (Figures 1F–1I).

### Setup of the perfusion system

The average blood pressure of a mouse is 120/102 mmHg, which corresponds to a mean arterial pressure (MAP) of approximately 112 mmHg.^4^ Since we want to flush the tissue thoroughly during perfusion without destroying the vascular system, it is crucial to flush the fluid through the animal’s body with the appropriate pressure. Hydrostatic pressure is used for this purpose. To calculate the hydrostatic pressure, following formula is used: h=P/p x g; where h: height of the bottle, P (pressure): desired pressure, p (density of the solution): 1000 kg/m^3^, g (acceleration due to gravity): approx. 9.81 m/s^2^.

To protect the tissue from damage, we use a pressure of 102 mmHg. This must be converted to pascals (Pa): 1 mmHg = 133.322 Pa, so 102 mmHg ≈ 13598.84 Pa. If we calculate this, we get h=13598.84 Pa/1000 kg/m^3^ × 9.81 m/s^2^ →h ≈ 1.39 m. Accordingly, the perfusion bottles have to be placed at a height of approximately 1.40 m above the animal. For this purpose, we recommend an infusion stand with a height of at least 2.20 m, which results in a working height of 45 cm with a perfusion table height of approximately 5 cm and a bottle height of 30 cm. If there are concerns about handling bottles with liquid in a height of 2.20 m or more, we would like to point out that the use of a peristaltic pump is also possible.

**Figure 2:**
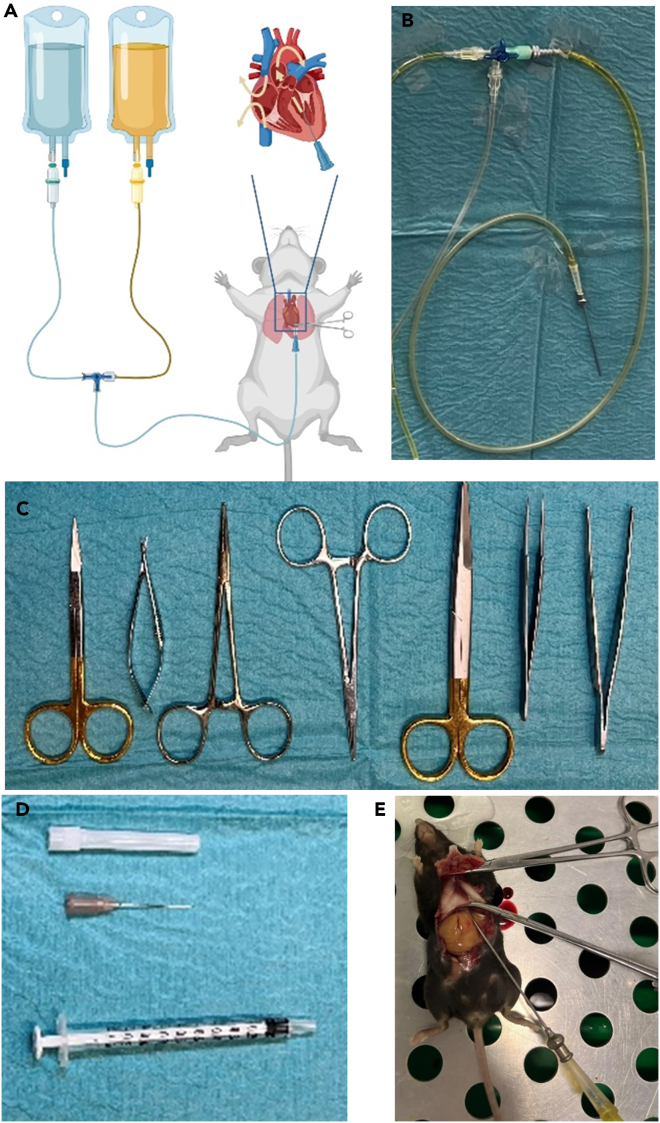
Setup of the perfusion system and preparations utensils (A) Schematic drawing of the perfusion system setup. (B) Mounting of the perfusion needle to the perfusion tubing. (C) Preparations utensils. (D) Syrings and needle tip. (E) Animal prepared for perfusion. Perfusion needle is mounted to the perfusion setup and secured with a needle holder in the left ventricle.

For perfusion, both infusion bottles have to be equipped with a perforated Intrafix system. It is important to note that the orange locking system must be pulled to the end of the tube, otherwise it cannot be operated later. Both Intrafix systems are then connected to a 3-way tap (Figure 1J). The remaining port of the 3-way tap has to be connected with a tube taken from a third Intrafix System after removing the filter system with a pair scissors to the Pravaz needle via a 3-way tap (Figure 2). If necessary, an extension tube can be used for this purpose (Figure 2B).

### Preparation utensils

For perfusion and subsequent preparation, we recommend the following instruments:

A coarse pair, a medium pair, and a fine pair of scissors, two clamps or needles, a coarse and a fine forceps (Figure 2C). In addition, a 1 mL syringe and a 26G needle tip are needed.

## Step-by-step method details

### Transcardial perfusion of a mouse

**Timing: About 20 min**

This step is necessary to flush blood out of the vasculature and to fix the tissue.

1. Anesthesia of the mouse with isoflurane.
  a. Place the mouse in the anesthesia chamber.
  b. Flush the chamber with vaporized isoflurane (4%).
  c. Wait until the mouse is under deep anesthesia.
  d. Sacrifice the mouse by cervical dislocation.
  e. Confirm the death of the animal.

***Note:*** Perfusion can also be performed under deep anesthesia.

This can be beneficial for the perfusion result. However, the differences in the perfusion result are minimal as long as one crucial factor is taken into account. The decisive factor is the timing of perfusion. Perfusion must be started before the blood begins to clot. To ensure this, heparin is used (Step 3 b) and perfusion should be started while the heart is still beating (Step 4). It should be noted that in certain countries, such as Germany, perfusion under anesthesia is considered animal experimentation and requires a high level of administrative effort, whereas this is not the case for perfusion after the killing of an animal.

**Figure 3:**
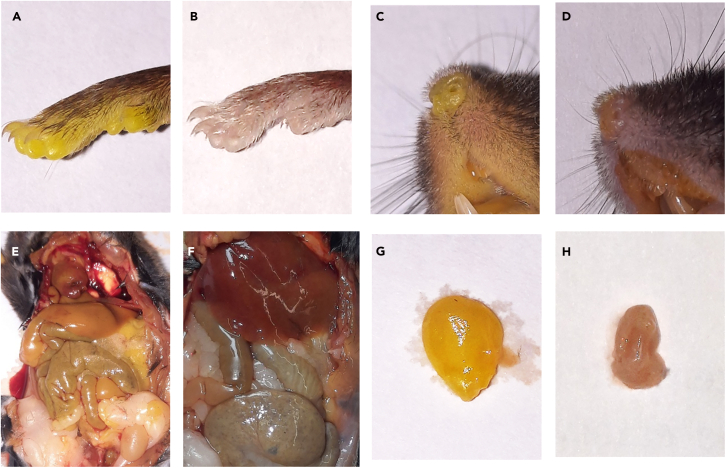
Perfusion and Fixation (A–H) Comparison of zamboni perfused (A; C; E; G) and unperfused (B; D; E; H) body parts; Paw (A and B); Nose (C and D); Abdomen (E and F); Urinary bladder (G and H).

2. Preparation of the animal.
  a. Place the animal on its back on to the perfusion table.
  b. Remove the fur from the thorax and open the abdomen with a longitudinal incision.
    ***Note:*** We recommend using coarse pairs of scissors and coarse forceps for this (Figure 2C).
  c. Carefully cut through the diaphragm with a few small incisions to ventilate the chest.
  d. Cut the ribs on both sides by making incisions along the lateral side.
  e. Cut down to approximately the level of the armpit and fold the thoracic shield upward until the heart is visible.
  f. Use a clamp or needle holder to fix it.
    ***Note:*** A still-beating heart supports perfusion.
3. Preparation for perfusion.
  a. Secure the heart with forceps.
  b. Inject 0.1 mL of heparin directly into the left ventricle using a 1 mL syringe and a 26G needle tip (Figure 2D).
    ***Note:*** This will prevent blood from clotting.
  c. Cut off the right auricle.
    ***Note:*** We recommend using medium pair or fine scissors for this (Figure 2C).
  d. Carefully open the left ventricle by making a small incision on the left side of the apex of the heart.
  e. Carefully insert the blunt perfusion needle, which is already connected to the perfusion system, into the left ventricle.
  f. Secure and seal the needle in the ventricle with a clamp or needle holder (Figure 2E).
    ***Note:*** Preparation is shown in Methods Video S1 and S2.
4. Perfusion with pre-rinse solution.
  a. Place the 3-way stopcock on the bottle containing the pre-flush solution.
    ***Note:*** Bottle should be suspended approximately 1.40 m above the animal.
  b. Open the orange locking system.
    ***Note:*** The hydrostatic pressure now pushes the pre-rinse solution through the left ventricle and the connected aorta into the animal's body.
  c. The blood leaves the body via the right atrium.
    ***Note:*** It is important to check that the solution is flowing continuously. If this is not the case carefully move the perfusion needle.
  d. Collect the blood and pre-rinse solution in the square plastic tub or tray of the perfusion table.
    ***Note:*** Blood and pre-rinse solution can later be disposed together with excess perfusion solution. Pre-rinsing is complete as soon as only clear fluid flows out of the right atrium. Perfusion with pre-rinse solution is shown in Methods Video S3 and S4.
5. Perfusion with fixative solution.
  a. Set the 3-way stopcock on the bottle containing fixation solution.
  b. Close the orange locking system for the pre-rinse solution.
  c. Open the orange locking system for the fixation solution.
    ***Note:*** The hydrostatic pressure now pushes the fixation solution into the animal's body and displaces the pre-rinse solution.
  d. Ensure that fixation is successful.
    ***Note:*** The fixation is successful and complete when the following criteria are met:
    i. Only yellow fluid leaves the right atrium.
    ii. Limbs and body become stiff.
    iii. Paws and skin become yellowish (Figures 3A–3E).
    iv. Liver changes color to pale yellow (Figures 3E and 3F).
      ***Note:*** Perfusion with Zamboni fixation solution is shown in Methods Video S4 and S5.

Troubleshooting 1. It should be noted that working with Zamboni can produce fumes that are harmful (Supplementary Information 1).

Methods Video S1. Preparation; side view, related to step 2

Methods Video S2. Preparation; top view, related to step 2

Methods Video S3. Pre-rinse solution; side view, related to step 3

Methods Video S4. Pre-rinse solution and fixation, related to step 3 and 4

Methods Video S5. Fixation, related to step 4

### Preparation and organ removal of male and female animals

**Timing: About 20 min**

This step is to harvest the organs of interest and to post-fixate the tissue.

**Figure 4:**
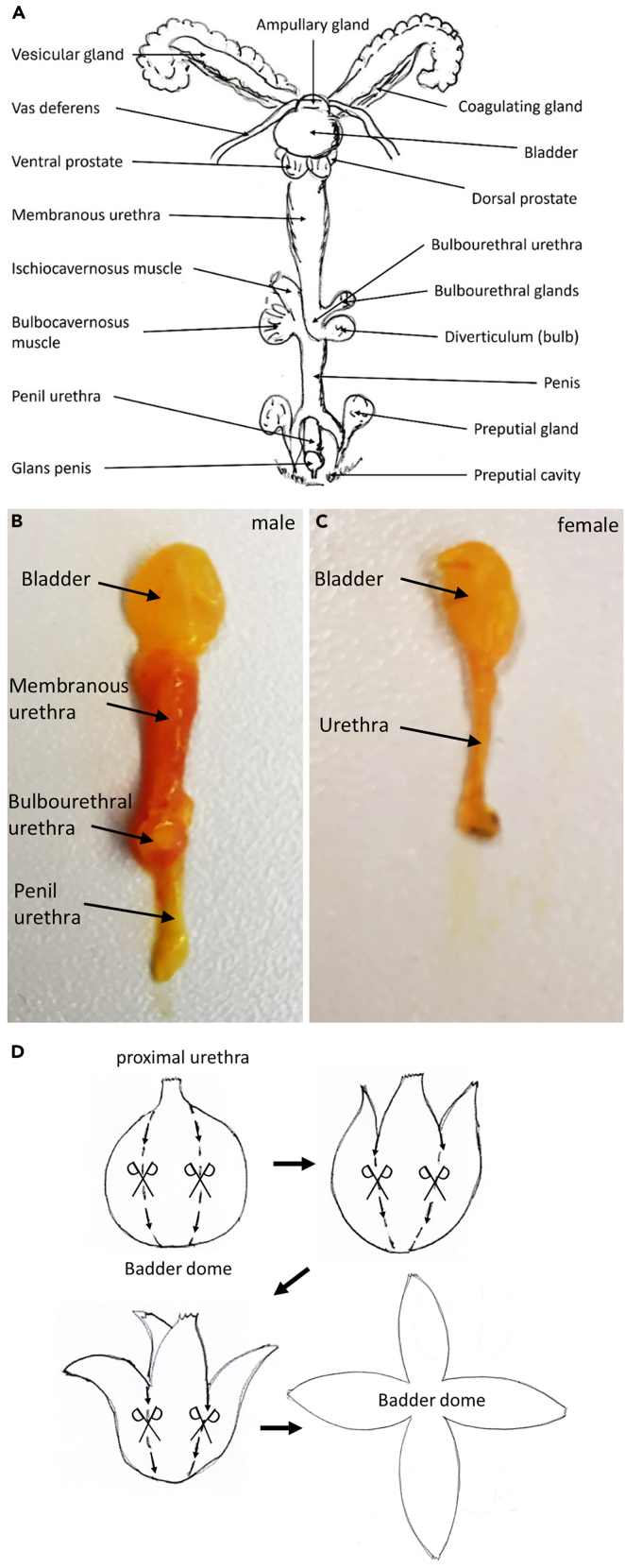
Urinary bladder and Urethra (A) Schematic drawing of the male urinary system adapted from Biology of the Laboratory Mouse5. (B) fixed male urinary bladder and urethra. (C) Fixed female urinary bladder and urethra. (D) Cutting template for opening a urinary bladder.

6. Preparation of urinary bladder and urethra from male and female mice.
  a. Place the mouse on its back on a dissection pad and secure it in place.
    ***Note:*** We recommend using a dissecting microscope.
  b. Remove any remaining fur and visceral fat covering the urinary bladder and the pubic bone.
  c. Push the abdominal organs carefully to one side to have access to the preparation field.
    ***Note:*** The urinary bladder and the beginning of the proximal urethra should now be freely accessible.
    i. In male animals: Cut the penis out of the surrounding tissue.
    ii. Remove the foreskin carefully by making an incision and then cutting it off with a circular cut along the base of the glans penis.
      ***Note:*** This also removes the preputial gland.
    iii. In female animals: Locate the external urethral orifice and carefully cut it out.
  d. Remove the tissue covering the pubic symphysis to locate and expose the bone.
  e. Severe the pelvic bone adjacent to the symphysis with an incision on both sides.
  f. Remove the symphysis with forceps.
    i. In male animals: Remove all glands (vesicular gland, ampullary gland, coagulating gland, dorsal and ventral prostate and the vas deferens).
      ***Note:*** The membranous urethra is connected to the urinary bladder and can move freely.
    ii. Remove glands and muscle surrounding bulbourethral urethra by making careful incisions.
      ***Note:*** The bulbourethral urethra is located next to the diverticulum (bulb), and the bulbourethral gland is surrounded by muscles (bulbocavernosus muscle and ischiocavernosus muscle).
    iii. Extract the penile part of the urethra from the penis by grasping it with forceps below the glans penis and separating it from the penis by lifting the urethra and cutting it free with small, careful incisions.
      ***Note:*** The penile urethra is located at the dorsal side of the penis. It should be noted that in the glans penis the urethra is firmly attached to the mouse's penile bone, making a neat preparation difficult. For orientation use the schematic drawing adapted from Biology of the Laboratory Mouse^5^ (Figures 4A and 4B). Preparation of a male mouse is shown in Methods Video S6.
    iv. In female animals: Remove the urethra completely by lifting the bladder with forceps and carefully separating the urethra from the surrounding tissue through small incisions (Figure 4C).
      ***Note:*** Female urethra is located ventral to the vagina. Preparation of a female mouse is shown in Methods Video S7.

Methods Video S6. Preparation of male mice, related to step 6

Methods Video S7. Preparation of female mice, related to step 6

### Handling of organs after removal

**Timing: 3–5 days**

This step is to post fix the tissue and remove the entire fixating solution.

7. Immediately after dissection, immerse the organs promptly in 50 mL fixative solution in a 50 mL falcon tube and allow them to post-fix for 2 h at 21°C in the dark.
8. Wash the tissue and remove the fixating solution.
  a. Replace the fixative solution with 50 mL 0.1 M phosphate buffer.
  b. Wash the organ on a shaker in the dark at room temperature.
  c. Replace puffer as soon as it turns yellow.
  d. Repeat step 2 and 3 until yellowish coloration disappears.
    ***Note:*** The more frequently the wash buffer is changed, the faster the fixative is washed out. Usually, 3 days are sufficient.
  e. Cut the urinary bladder open.
    ***Note:*** It is very difficult to do so after clearing due to the high transparency of the tissue.

We recommend cutting the bladder open with two cross-shaped incisions from the apex (Figure 4D).

### Hydrogel embedding and delipidation for tissue clearing

**Timing: 1–4 days**

This step is for polymerization of tissue components with hydrogel monomers for stabilizing proteins and tissue structure and to solubilize lipids in the tissue and to remove them.

9. Polymerization of tissue components.
  a. Incubate samples 8 h in 50 mL hydrogel solution in a 50 mL falcon tube at 4°C in the dark.
    **CRITICAL:** Hydrogel solution should be prepared fresh prior to the experiment. To prevent the hydrogel solution from polymerizing to early, pre-cooled buffer should be used to prepare the solution.
  b. Polymerize Hydrogel solution at 37°C in a shaking water bath until it becomes viscous.
    ***Note:*** This usually takes 4 to 5 h.
  c. Wash samples in 50 mL of 0.1 M phosphate buffer in 50 mL falcon tube for 2 h with gentle shaking at room temperature in the dark.
    ***Note:*** Insufficient polymerization may cause poor preservation of the tissue structure. Troubleshooting 2.
10. Delipidation.
  a. Incubate samples in 50 mL 8% SDS solution in a 50 mL falcon tube for desired time at 37°C in shaking water bath in the dark.
    ***Note:*** The time required for delipidation must be determined empirically. Normally 2–3 days are sufficient. 8% SDS solution can be prepared in advance and stored for at least 6 months at room temperature. If stored below 20°C, SDS may precipitate.
  b. Wash samples in 50 mL 0.1 M phosphate buffer in a 50 mL falcon tube at room temperature in the dark to remove SDS.
    ***Note:*** Change phosphate buffer several times until no more foam is visible in the washing solution. The more frequently the wash buffer is changed, the faster the SDS is washed out. Normally 2 to 3 days should be sufficient. Troubleshooting 3.

### Immunohistochemistry

**Timing: 5 days**

This step is to label proteins/peptides with antibodies.

11. Labelling proteins by immunohistochemistry.
  a. Incubate samples 8 h in PBS with 10% horse serum, 0.5% Tween 20, 0.1% bovine serum at 21°C in the dark to block nonspecific binding sites.
    ***Note:*** The amount of blocking solution depends on the size of the organ. Use enough blocking solution to completely cover the whole organ.
  b. Wash samples in 50 mL 0.1 M phosphate buffer in a 50 mL falcon tube for 2 h at room temperature.
  c. Incubate samples with primary antibodies in desired concentration for about 3 days at room temperature in the dark by gentle shaking.
    ***Note:*** The amount of antibody solution and the duration of incubation time depends on the size of the organ and have to be determined empirically. For an initial trial, use the antibody concentration that also works in conventional cryosections. Incubation time depends highly on the antibody format used. Smaller antibody formats are recommended. Penetration depth of a full IgG is about 500 μm within 3 days. Antibody solution should cover samples completely.
  d. Wash samples with 50 mL 0.1 M phosphate buffer in a 50 mL falcon tube for 3 days at room temperature in the dark by gentle shaking. Change phosphate buffer several times.
  e. Incubate samples in secondary antibody solution for 48 h at room temperature in the dark by gentle shaking.
    ***Note:*** The same requirements apply to the secondary antibody as to the first.
  f. Wash samples with 50 mL 0.1 M phosphate buffer in a 50 mL falcon tube for 48 h at room temperature in the dark by gentle shaking.
    ***Note:*** Change phosphate buffer several times.
  g. Place the samples in refractive index matching solution (RIMS) for 2 days in the dark at room temperature.
    ***Note:*** Use at least twice the organ’s volume.
  h. Store the samples, now ready to be imaged by confocal laser scanning microscopy, in RIMS at 4°C in the dark.
    ***Note:*** Since RIMS is very hygroscopic the storage chamber must be airtight, otherwise streaks will form.
    ***Note:*** Controls that should be included to ensure the specificity of the antibodies.

Primary antibody controls: Western blot and preabsorption. By western blot analysis it is possible to check whether an antibody recognizes a protein of the expected molecular weight. For this purpose, an organ/tissue should be chosen that is known to express the protein of interest. To check for the binding specificity of the first antibody, a preabsorption experiment is highly recommended. For this purpose, prior to application, the antibody solution is incubated with a peptide/protein against which the antibody is directed. The concentration of the desired peptide/protein must be determined empirically. Preabsorption is helpful in immunohistochemistry as well as in western blot experiments. In such a preabsorption experiment, ideally no signal should be detectable.

Secondary antibody control: To investigate whether the second antibody generates non-specific signals, it is advisable to conduct an experiment in which the primary antibody is omitted. Ideally, no signal should be observed in this experiment.

### Imaging with confocal laser scanning microscopy

**Timing: 1–5 h**

This step is to produce images and to provide z-stacks for 3D rendering and data analysis.

**Figure 5:**
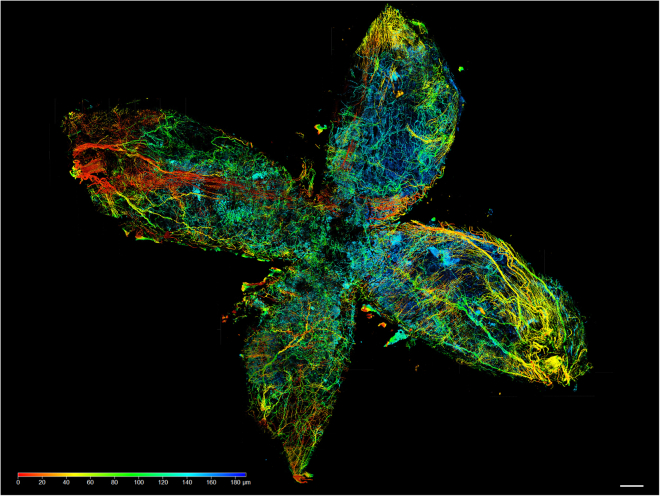
Cleared urinary bladder Cleared urinary bladder labeled with anti-Tyrosine Hydroxylase antibody imaged by confocal laser scanning microscopy. The bladder was opened with two cross-shaped incisions from the apex. Scale bar 800 μm.

**Figure 6:**
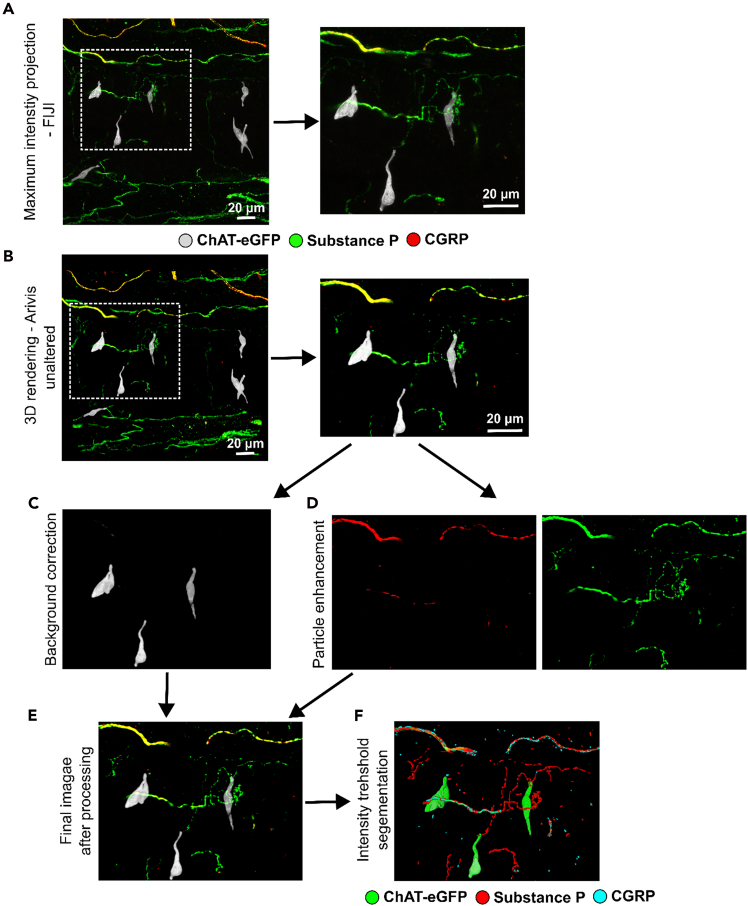
Workflow of image processing (A) original pictures are processed in Fiji-ImageJ (NIH), maximum intensity projection, left panel whole imaged area, right panel enlargement of region of interest. (B–F) Image processing using in Arivis Vision Pro (Version 4.1.2, Zeiss). (B) Dataset was imported in Arivis and 3D projection created using the maximum mode, left panel whole imaged area, right panel enlargement of region of interest. (C) Background correction using a discrete gaussian function for channel 1 (FITC). (D) Background correction using a discrete gaussian function for channel 2 (Cy3) and channel 3 (Cy5), followed by particle enhancement in both channels. (E) final merged and processed image. (F) Optional intensity threshold segmentation using the simple method.

12. Preparing slides and producing z-stack Images.
  a. Prepare glass slides with sample wells using appropriate spacers.
  b. Place samples inside the well.
  c. Easily overfill the well with RIMS.
    ***Note:*** Take care not to induce air bubbles.
  d. Place a cover slip on top of the well. Again, avoid sealing air bubbles in the well.
  e. Remove any overflow RIMS with a paper towel.
  f. To acquire images, we used a confocal microscope (LSM 710 or Stellaris 5) equipped with a 10× objective (10×/0.3 Figure 5) or a 63× objective (Plan-Apochromat 63×/1.40 oil DIC M27 Figure 6).
    ***Note:*** To generate Figure 6 z-stack consisted of 40 slices and a total range of 38 μm was acquired. The image size was 224.92 × 224.92 μm, with a pixel size of 0.11 × 0.11 × 0.97 μm. For optimal 3 D rendering we recommend a z-step size of about 3× times xy pixel size. The acquisition modalities are depicted in Table S1. Optimal imaging conditions have to be determined for each sample. Troubleshooting 4.

### 3D rendering of confocal z stacks to produce 3D movies

**Timing: 1–5 h**

**Figure 7:**
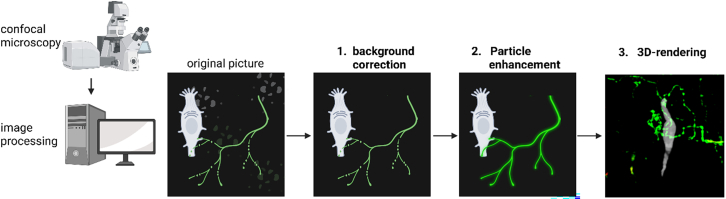
Schematic overview of the image processing workflow Schematic drawing shows imaging processing workflow from original picture to 3D-rendering.

This step is to transform the acquired two-dimensional z-stacks into three-dimensional projections (workflow Figure 7).

***Note:*** For image processing, we utilized the software Arivis Vision Pro. Alternatively, freely available software such as ImageJ (NIH) can be used, though with certain limitations.

13. Images processing.
  ***Note:*** In the original dataset, some background fluorescence is evident (Figures 6A and 6B; Methods Video S1). The nerve fiber terminals labeled with substance P and CGRP appear punctuated and thin, which makes it challenging to trace their direction, therefore the imaging steps described below were done. Imaging processing parameters are shown in Table S2.
  a. Import z-stack dataset in Arivis and create a 3D projection using the maximum mode, left panel whole imaged area, right panel enlargement of region of interest (Figures 6B and 7).
  b. Perform background correction using a discrete gaussian function (Figures 6C and 7) to reduce background signals.
    ***Note:*** Background signals may result from tissue autofluorescence.
  c. Perform particle enhancement.
    ***Note:*** This step increases the contrast of structures of a specified dimension, like punctuated and thin nerve fibers. Thereby making the nerve fiber easier to track and segment subsequently (Figures 6D and 7). Similar image processing can be performed in ImageJ/FiJi using the process >>Filters >>Gaussian Blur (5 sigma radius) and process >>Filters >>TopHat (2 pixel respectively) (Figures 6A and 7). The 3D visualization performed with FiJi requires the 3D viewer plugin and is sufficiently different from Arivis, the reader is encouraged to refer to the manual for that plugin.
  d. Merge final image (Figures 6E and Methods Video S8).
  e. Process image.
    ***Note:*** The final image can optionally undergo further segmentation into objects by applying an intensity threshold segmentation function. This method enables discrimination of specific objects based on their fluorescence intensity. The segmented objects can then be analyzed further, such as for tracking of nerve fibers (Figures 6F and Methods Video S9). Optimal imaging conditions must be determined for each sample. All parameters used in the described image processing workflow are listed in Table S2.

Methods Video S8. 3D Rendering, related to step 13

Methods Video S9. Intensity threshold segmentation, related to step 13

## Expected outcomes

Successful perfusion results in well-fixed tissue. At this step, it is possible to freeze the organ in liquid nitrogen and prepare cryosections or embed the organ in paraffin and prepare paraffin sections. For cryosections, we recommend transferring the washed specimens to 18% sucrose (Carl Roth) in 0.1 M phosphate buffer and incubation for 8 h at 4°C. The specimens are then embedded in Tissue-Tek® O.C.T.™ Compound (Sakura Finetek USA, Inc., USA) and flash frozen in liquid nitrogen. We recommend a section thickness of 10 μm prepared using a microtome e.g., HM560 cryostat (Thermo Fischer Scientific). For paraffin sections, the tissue is embedded in paraffin and cut into 5 μm thick sections. Before antibody treatment, the sections should be treated with 0.005 M phosphate buffer with 10% horse serum (Sigma Aldrich), 0.5% Tween 20 (Sigma Aldrich), and 0.1% bovine serum albumin (BSA; Sigma Aldrich) for 1 hour to saturate non-specific protein binding sites.

In this protocol we instead clear whole organs. This provides the possibility of three-dimensional analysis of labeled structures. By removing lipids and the adjustment of the refractive index by the use of histodenz, light-scattering, reflection and absorption caused by tissue components are reduced. The tissue becomes transparent without destroying its spatial structure. It also allows antibodies to penetrate deeper into the tissue, enabling uniform and complete staining of even thick samples. This leads to higher image quality and allows fluorescence or confocal microscopy deeper into the tissue.

With this technique, it is more feasible to examine the detailed three-dimensional orientation, obtain a comprehensive overview of innervation and distribution of cells and other structures of interest throughout of the whole organ. Such an overview is not possible in 2D. Although it is possible to examine a detailed view of a single cell and its interaction with nerve fibers, finding such a combination in a section is very time-consuming, labor-intensive, and costly, as an enormous number of sections must be stained and examined. This is evident in cleared tissue, as the whole tissue can be examined at once and regions of interest scanned in high resolution.

In our samples, neuronal network of the urinary bladder (Figure 5) and the urethra (refer to Schmidt et al. 2025^1^) as well as the topographical proximity between nerve fibers and rare tuft cells (Figure 6; Methods Video S8, refer to Schmidt et al. 2025^1^) are investigated and shown. Three-dimensional images can be produced by optical clearing of whole organs in combination with imaging techniques like high resolution confocal microscopy. For three-dimensional rendering of acquired confocal microscopy z-stack images, the image analysis software Arivis Vision Pro was employed. A 3D rendered dataset offers several advantages over conventional maximum intensity projections (MIP) or single-plane images. In the current dataset, the primary objective was to determine whether tuft cells are in close proximity to peptidergic nerve fibers. Conventional MIP generate only two-dimensional images, which lack spatial information; thus, it is not possible to distinguish whether a nerve fiber is located behind the cell of interest or in close contact with it.

Overall, the combination of optimized tissue fixation, clearing, and data collection and analysis significantly improves the outcome of immunohistochemical analysis.

## Limitations

Here we describe a sequence for tissue fixation, sample processing and evaluation which offers the possibility to study samples three-dimensionally and in greater depth. However, this also has some disadvantages and limitations. Tissue fixation represents a particularly critical step. A major challenge is the even distribution of the fixing solution: Insufficient flow or air bubbles may cause uneven fixed tissue. Especially in larger specimens or poorly vascularized structures, this may cause inhomogeneous fixation. Biological variability, such as differences in the vascular system within individuals, may also affect reproducibility. Additionally, perfusion is technically demanding and requires experience to avoid damage to the blood vessels and pressure fluctuations that could lead to leakage within the vascular system.

Next, clearing is a time-consuming process and most of the incubation times have to be determined empirically. This is especially important if reporter mice are used, since longer incubation times can lead to a loss of fluorescence intensity. If antibodies are used, special attention must be paid to the clearing process since the used reagents may damage or mask antigen structures.

Finally microscopic data analysis by confocal laser scanning microscopy (CLSM) is also challenging. Light-scattering, reflection and absorption within the cleared samples are reduced but not completely gone, making the examination of larger samples particularly difficult. As already mentioned, light exposure during sample preparation or prolonged laser exposure may cause fluorophores to fade, which impairs signal intensity and image quality. Differences in the refractive index between the clearing medium, objective, and immersion medium can also lead to image distortion or blurring. Another limiting factor is the high technical and computational effort involved: The acquisition of large-volume, high-resolution data sets generates considerable amounts of data, the processing and analysis of which is time-consuming and places high demands on computer performance. In addition, CLSM is relatively slow compared to other imaging techniques, such as light sheet microscopy, which results in long scanning times and an increased risk of phototoxic effects. Depending on tissue thickness, size of the sample or large areas of interest, more developed imaging modalities such as multiphoton or light sheet microscopy can be necessary. Regarding the 3D rendering we usually would recommend a z-step size of 3× times xy pixel size, which in our sample was a slight undersampling. It is important to note that these 3D rendering impose certain limitations on the analysis of specific parameters. For instance, measuring fluorescence intensity of structures is not feasible after the processing steps, since they were artificially altered.

## Troubleshooting

### Problem 1

Uneven distribution of the perfusion solution. (related to step 4 and 5).

This can occur if the vascular system is not fully intact or if air bubbles, clots, or vessel ruptures obstruct the flow. As a result, certain tissues or organs may not be adequately reached by the fixative leading to insufficient fixation. This uneven penetration can negatively affect sample structure, staining efficiency, and overall image quality in subsequent imaging steps.

### Potential solution

• Preparation of the vascular system: Before perfusion with fixative solution, blood should be removed. Formation of blood clots that might obstruct the flow should be prevented by heparin injected into the left ventricle. Perfusion with pre-rinse solution helps to keep vessels open and improve fixative penetration. It is important to ensure that only pre-rinse solution flows from the opened right atrium of the heart.
• Optimization of perfusion pressure and flow rate: Excessive pressure can damage the blood vessels, while low pressure results in insufficient tissue perfusion. The perfusion pressure should approximate the animal’s physiological blood pressure (e.g., about 100 mmHg in mice). That is why it is important to ensure that the infusion bottles are positioned at the correct height and perfusion speed could be controlled by the orange locking system.
• Use of temperature-controlled solutions: Fixatives and pre-rinse solution should be freshly prepared and pre-warmed to body temperature to enhance flow and chemical reactivity.
• Accurate cannulation: The blunt perfusion needle must be precisely positioned (in the left ventricle for mice) to ensure even distribution through the flushed vascular system. A free outflow from the right atrium should be maintained to allow continuous flow rate. If there is a leak on the side of the left ventricle, the seal should be replaced to ensure that the needle is positioned correctly.
• Verification of perfusion efficiency: The yellow color of Zamboni helps to confirm successful perfusion. This allows assessment of solution distribution throughout the body. If there is no yellowing of the paws, organs, and tip of the nose, the perfusion is unsuccessful or incomplete.

### Problem 2

Formation of tissue-hydrogel matrix (related to step 9).

Insufficient polymerization of tissues with hydrogel monomers may cause poor preservation of structural and biochemical properties of tissues.

### Potential solution

Polymerization of the hydrogel monomers is most effective at 37°C. To prevent premature polymerization of the hydrogel monomers at room temperature, the solution should be prepared at 4°C. It is important to ensure that the hydrogel monomer solution is always freshly prepared. To guarantee the best possible structural preservation of the organs during delipidation, the organs should be incubated in the hydrogel monomer solution for at least 12 h at 4°C. Polymerization then takes place at 37°C in a water bath. After approximately 3–4 h with gentle shaking, the polymerization should be complete. This can be easily recognized by the viscosity of the hydrogel solution.

### Problem 3

Organ delipidation with SDS (related to step 10).

Delipidation is a critical step for further sample processing, i.e., antibody treatment. Excessive cleaning can destroy the structural and biochemical properties of the tissue, while insufficient cleaning can result in poor antibody penetration into the tissue.

### Potential solution

The time required for delipidation must be determined empirically for each organ. For the organs used here, 2 to 3 days of delipidation are generally sufficient. The optimal clearing time is reached when the organ begins to become transparent.

### Problem 4

Confocal imaging (related to step 12).

Improper handling of the cleared organs may negatively affect data acquisition by confocal imaging.

### Potential solution

To obtain optimal results, data collection must be performed in RIMS solution. Since RIMS is very hygroscopic, care must be taken to ensure the sample chamber is airtight, otherwise streaks will form. To prevent organ movement during data collection, the sample chamber should be as small as possible.

### Problem 5

3D Rendering (related to step 13).

3D rendering is not possible because the Z stack does not provide enough data information.

### Potential solution

Repeat step 12 and decrease the Z step size to obtain more images. For optimal 3D representation, we recommend a Z step size that is approximately three times the XY pixel size.

## Resource availability

### Lead contact

Further information and requests for resources and reagents should be directed to and will be fulfilled by the lead contact, Klaus Deckmann (klaus.deckmann@anatomie.med.uni-giessen.de).

### Technical contact

Technical questions on executing this protocol should be directed to and will be answered by the technical contacts, Uwe Pfeil (uwe.pfeil@anatomie.med.uni-giessen.de) or Klaus Deckmann (klaus.deckmann@anatomie.med.uni-giessen.de).

### Materials availability

This study did not generate new unique reagents.

### Data and code availability

The microscopy datasets supporting the current study have not been deposited in a public repository because of their large file size but are available from the corresponding author on request.

## Acknowledgments

We thank Martin Bodenbenner for technical assistance. This work was supported by the 10.13039/501100001659German Research Foundation (10.13039/501100001659DFG, 544054869 to K.D.). The graphical abstract and figures were created in https://BioRender.com.

## Author contributions

U.P., F.D., A.P., P.V.A., P.M.L., and K.D. performed the experiments and analysis. K.D. obtained funding. U.P., F.D., A.P., P.V.A., L.B., P.M.L., and K.D. drafted the manuscript. This work was supervised by L.B. and K.D.

## Declaration of interests

The authors declare no competing interests.
