# Supplementary material for: Preparation of cleared whole-mount urethra and urinary bladder from transcardially perfused mice for immunolabeling and analysis by CLSM
Source: STAR Protoc. 2026 May 7;7(2):104542. doi: 10.1016/j.xpro.2026.104542 (PMC13186176; doi:10.1016/j.xpro.2026.104542)
Supplement: Document S1. Figure S1 and Tables S1 and S2 [file mmc1.pdf]

Figure S1: Figure 6 for reader with red-green color blindness in a version with cyan and magenta.

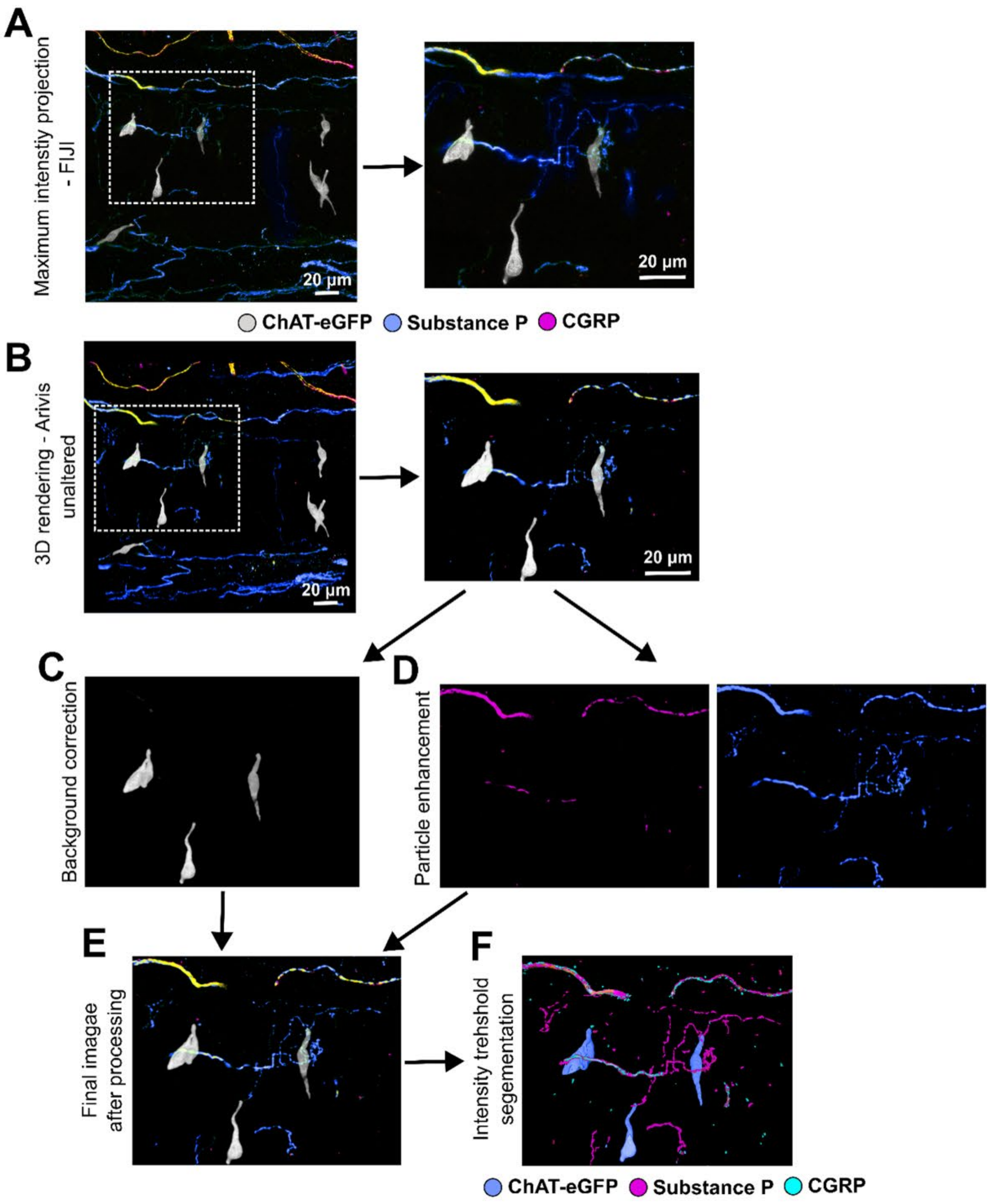

Table S1: Acquisition modalities for confocal laser scanning microscopy. Caption for table with contents.

Modalities	Channel 1	Channel 2	Channel 3
Fluorophore	FITC	Cy3	Cy5
Target	GFP	Substance P	CGRP
Pinnhole	61 µm	84 µm	85 µm
Pixel dwell time	0.64 µs	0.64 µs	0.64 µs
Excitation wavelength	488	561	633
Laser power	17.7	2.3	21.3
Averaging frame	2	2	2
Detection range	496-546 nm	555-605 nm	638-708 nm
Gain	582	621	732

Table S2: Imaging processing parameters

Step	Method	Modalities	Channel 1	Channel 2	Channel 3
		Fluorophore	FITC	Cy3	Cy5
		Target	GFP	Substance P	CGRP
Background correction	Discrete Gaussian	Diameter	5	Not performed	Not performed
Particle enhancement		Strength	Not performed	1.2	1.2
		Diameter		2	2
Segmentation	Intensity treshold segementation, simple, bright	Threshold	14	10	3

Supplementary Information S1: Safety data sheet.

Picric Acid

APPLICATION:

Picric Acid, also referred to as 2,4,6-trinitrophenol, carbazotic acid, phenol trinitrate, or picronitric acid, is commonly used in tissue fixative solutions and serves as a component in various specialized staining procedures.

Picric Acid can be handled safely when kept hydrated and stored in an aqueous solution containing at least 30% water. A significant explosion risk exists if Picric Acid becomes dry or partially dehydrated, is exposed to heat, or comes into contact with metals or metallic salts.

1. Do not dispose of Picric Acid solutions via sink drains or sanitary sewer systems.
2. Collect all Picric Acid waste solutions in a properly labeled, leak-proof container for appropriate disposal.
   a. Do not use metal containers.
   b. Do not use containers with metal caps or lids.

3. Thoroughly rinse tissues fixed with Picric Acid (e.g., Bouin’s fluid, Hollande fixative, Zamboni fixative) before placing them into a tissue processor to prevent residual Picric Acid from entering the processing system.
   a. Recommendation: Rinse tissues extensively under running tap water, followed by a wash of at least 15 minutes in 70% ethanol.

Picric Acid is classified as a toxic substance and a health hazard. Appropriate personal protective measures must be followed during handling. The associated safety risks are detailed in the product’s Safety Data Sheet (SDS) and should be carefully reviewed and assessed by the user.

Dispose of Picric Acid solutions in accordance with all applicable local, state, and federal regulations.

REFERENCES:

Dapson, Janet Crookham, and Richard Dapson. *Hazardous Materials in the Histopathology Laboratory: Regulations, Risks, Handling, and Disposal*. 4th ed. Battle Creek, MI: Anatech, 2005.

https://ehs.wisc.edu/wp-content/uploads/sites/1408/2020/08/CHM-GUI-006-NEW.pdf

http://nj.gov/health/eoh/rtkweb/documents/fs/1946.pdf

Zamboni Fixative

Zamboni Fixative is a phosphate-buffered picric acid–formaldehyde (PAF) fixative designed for applications in light and electron microscopy. Zamboni Fixative ensures rapid penetration, effective general fixation, and optimal preservation and stabilization of cellular proteins.

METHOD:

Fixation:
• Small biopsies: Minimum 1 hour
• Larger biopsies: Minimum 4 hours

FIXATION PROCEDURE:

1. Place fresh tissue into Zamboni Fixative immediately after surgical excision.

2. Keep tissue specimens in Zamboni Fixative until processing.
   (Tissue specimens may be stored indefinitely in Zamboni Fixative at room temperature without loss of preservation quality)

3. Thoroughly rinse Zamboni-fixed tissue in running tap water, followed by Phosphate Buffered Saline 0.1M, pH 7.4 for a minimum of 15 minutes prior to processing.

4. Processing:
   a. **Light microscopy:** Transfer to a tissue processor, beginning either with the Formalin 10%, Phosphate Buffered (1090) fixation step or the first dehydration station.
   b. **Electron microscopy:** A secondary fixation with osmium tetroxide is recommended. Refer to the appropriate electron microscopy processing protocol.

Safety Data Sheet

1. PRODUCT

Product Name: Zamboni Fixative

2. HAZARD(S) IDENTIFICATION

2.1. Classification of the substance or mixture
GHS Classification, (in accordance with 29 CFR1910.1200)
Skin sensitisation, Category 1
Serious eye damage, Category 1
Skin irritation, Category 2
Carcinogenicity, Category 2
2.2. GHS Label elements
Signal Word DANGER

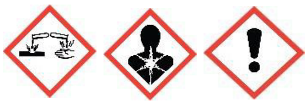

Hazard Statement(s):

- May cause an allergic skin reaction
- Causes serious eye damage
- Causes skin irritation
- Suspected of causing cancer

Precautionary Statement(s):

Prevention:

- Obtain special instructions before use.
- Do not handle until all safety precautions have been read and understood.
- Do not breathe dust/fume/gas/mist/vapours/spray.
- Contaminated work clothing should not be allowed out of the workplace.
- Wear protective gloves/protective clothing/eye protection/face protection.

Response:

- IF ON SKIN: Gently wash with plenty of soap and water.
- Take off contaminated clothing and wash before reuse.
- If skin irritation or a rash occurs: Get medical advice/attention.
- IF IN EYES: Rinse cautiously with water for several minutes. Remove contact lenses if present and easy to do – continue rinsing.
- Specific treatment: see first aid measures in section 4.
- Immediately call a POISON CENTER or doctor/physician.

Storage:

- Store locked up.

Disposal:

- Dispose of contents/ container to an approved waste disposal plant.

2.3 Description of any hazards not otherwise classified None

2.4 >1% of mixture with unknown acute toxicity None

3. COMPOSITION/INFORMATION ON INGREDIENTS

3.2 Mixture

Hazardous Components

Components		Concentration
Name	Paraformaldehyde	2%
CAS-No.	30525-89-4	
Name	Picric Acid	15%
CAS-No.	1310-73-2	

4. FIRST-AID MEASURES

4.1 Description of necessary measures

Inhalation (breathing)

IF INHALED: Remove victim to fresh air and keep at rest in a position comfortable for breathing.

Immediately call a POISON CENTER or doctor/physician

Skin Contact

IF ON SKIN (or hair): Remove/Take off immediately all contaminated clothing. Rinse skin with water/shower. Wash contaminated clothing before reuse. Immediately call a POISON CENTER or doctor/physician.
Eye Contact
IF IN EYES: Rinse cautiously with water for several minutes. Remove contact lenses if present and easy to do – continue rinsing. Immediately call a POISON CENTER or doctor/physician
Ingestion (swallowed)
IF SWALLOWED: Immediately call a POISON CENTER or doctor/physician. Rinse mouth.
4.2 Most important symptoms and or effects, acute and delayed
The most important symptoms/effects are presented in Section 2 and or Section 11.
4.3 Indication of any immediate medical attention and special treatment needed
No data available

5. FIRE-FIGHTING MEASURES

5.1 Suitable extinguishing media
Carbon dioxide, dry chemical, water spray, alcohol-resistant foam.
5.2 Specific hazards arising from the substance or mixture
No data available
5.3 Protective equipment and precautions for fire-fighters
Wear a positive-pressure self-contained breathing apparatus if necessary. Wear chemical resistant clothing as recommended by clothing manufacturer.

NFPA Rating
Health hazard: 2
Fire hazard: 1
Reactivity hazard: 0

6. ACCIDENTAL RELEASE MEASURES

6.1 Personal precautions, protective equipment and emergency procedures
Apply personal protective equipment (see Section 8). Use in a properly ventilated area. Avoid breathing vapors. Avoid skin contact. Avoid eye contact. Wash hands after use. In case of large spill, remove personnel to a safe area. Keep product away from heat, flame, ignition sources, and reactive materials. Avoid accumulation of vapor to form explosive concentration. Pay particular attention to low areas where vapor accumulates more easily.

6.2 Methods and material for containment and cleaning up
Apply personal protective equipment (see Section 8). Ensure proper ventilation. Contain spill. Prevent
further leakage if possible and safe to do so. Evacuate area and limit access. Prevent entry of material into sewage drains and confined areas. Dispose of any contaminated materials according to local regulations. Eliminate sources of ignition.

7. HANDLING AND STORAGE

7.1 Precautions for safe handling
Keep away from heat/sparks/open flames/hot surfaces – No smoking. Do not breathe dust/fume/gas/mist/vapours/spray. Wear protective gloves/protective clothing/eye protection/face protection.
7.2 Conditions for safe storage, including any incompatibilities
Refer to Section 2.2 for proper storage temperature. Store the tightly closed container in a cool, dry, well-ventilated area.

8. EXPOSURE CONTROLS/PERSONAL PROTECTION

8.1 Control Parameters
Components with limit values that require monitoring at the workplace

Component	CAS-No.	Regulatory	Value	Parameters
Paraformaldehyde	30525-89-4	OSHA PEL	TWA	0.75 ppm
		OSHA PEL	STEL	2 ppm
		NIOSH REL	STEL	0.016 ppm
		NIOSH REL	TWA	2 ppm
		ACGIH TLV	C	0.3 ppm
Picric Acid	88-89-1	OSHA PEL	TWA	0.1 mg/m^3 (skin)
		ACGIH TLV	TWA	0.1 mg/m^3 (skin)
		NIOSH REL	TWA	0.1 mg/m^3 (skin)
		NIOSH REL	STEL	0.3 mg/m^3 (skin)

8.2 Exposure Controls
Appropriate engineering controls
Use in a properly ventilated area. Remove/wash before reuse contaminated clothing. Wash hands upon exiting work premises. Use product in an appropriately designated fume hood. Take measures to keep concentrations below acceptable limits.

8.3 Personal Protective Equipment
Eye/Face protection
Wear chemical safety goggles and/or a full face shield if splashing is possible. Keep eye wash fountain nearby.
Skin Protection
Wear chemical-resistant gloves. Gloves should be resistant to components of product. Refer to glove manufacturer for appropriate type and glove thickness.
Body Protection
No data available
Respiratory Protection
Respirators should only be used if the employer has implemented a written program that takes into account workplace conditions, requirements for worker training, respirator fit testing, and medical exams, as described in the OSHA Respiratory Protection Standard (29 CFR 1910.134).
In case of emergency, entry into unknown concentrations, or escape, wear a self-contained positive-pressure breathing apparatus.

9. PHYSICAL AND CHEMICAL PROPERTIES

9.1 Information on basic physical and chemical properties

Physical state	
Odor	Yellow liquid
Odor threshold	Pungent odor
pH	No data available
Melting point/freezing point	No data available
Initial boiling point and boiling range	No data available
Flash point	No data available
Evaporation rate Flammability (solid, gas)	No data available
Upper flammability or explosive limits	No data available

Lower flammability or explosive limits	No data available
Vapor pressure	No data available
Vapor density	No data available
Relative density	No data available
Solubility(ies)	Water soluble
Partition coefficient: n-octanol/water	No data available
Auto-ignition temperature	No data available
Decomposition temperature	No data available

10. STABILITY AND REACTIVITY

10.1 Reactivity
No data available

10.2 Chemical stability
Stable in a closed container within label-specified storage temperature and expiration date.

10.3 Possibility of hazardous reactions
No data available

10.4 Conditions to avoid
Heat, sparks, open flame, and ignition sources.

10.5 Incompatible materials
Strong oxidizing materials, strong acids, strong bases, strong reducing agents, heavy metals, heavy metal salts, and ammonia.

10.6 Hazardous decomposition products
Paraformaldehyde decomposes slowly in water to form toxic and flammable formaldehyde gas.

11. TOXICOLOGICAL INFORMATION

11.1 Information on toxicological effects
Inhalation exposure
No data available

Oral exposure
No data available

Dermal exposure
No data available

Skin corrosion/irritation
No data available

Serious eye damage/irritation
No data available

Respiratory or skin sensitization
Paraformaldehyde may cause a skin allergy and an asthma-like allergy. Future exposure can cause asthma attacks with shortness of breath, wheezing, coughing, and/or chest tightness.

Germ Cell mutagenicity
No data available

Reproductive toxicity
No data available

Specific target organ toxicity - single exposure
No data available

Specific target organ toxicity - repeated exposure
Repeated high exposure to paraformaldehyde may affect the kidneys.

Aspiration hazard
No data available

Acute toxicity
Picric Acid:
LD50 rat 200 mg/kg

Carcinogencity
IARC: Formaldehyde a decomposition product is Group 1, carcinogenic to humans
NTP: Formaldehyde: Known human carcinogen
OSHA: Formaldehyde: Specifically regulated carcinogen

Additional information
RTECS: No data available

12. ECOLOGICAL INFORMATION

12.1 Ecotoxicity
No data available

12.2 Persistence and degradability
No data available

12.3 Bioaccumulative potential
No data available

12.4 Mobility in soil
No data available

12.5 Other adverse effects
No data available

13. DISPOSAL CONSIDERATIONS

13.1 Waste disposal methods
Contents
Dispose of contents in a safe manner to comply with local, state and federal regulations.
Contact alicensed professional waste disposal service to dispose of this material.

Contaminated packaging
Dispose of packaging in a safe manner to comply with local, state and federal regulations.
Contact a licensed professional waste disposal service to dispose of this material.
